# The Effectiveness of Community Occupational Therapy Interventions: A Scoping Review

**DOI:** 10.3390/ijerph18063142

**Published:** 2021-03-18

**Authors:** Maria-Francesca Estrany-Munar, Miguel-Ángel Talavera-Valverde, Ana-Isabel Souto-Gómez, Luis-Javier Márquez-Álvarez, Pedro Moruno-Miralles

**Affiliations:** 1 ASPAYM Baleares (Association of Spinal Cord Injuries and Other Physical Disabilities), 07006 Mallorca, Spain; estranymunarxisca@gmail.com; 2 Integra Saúde Research Unit, Department of Health Sciences, Universidade da Coruña, 15001 A. Coruña, Spain; 3 Integra Saúde Research Unit, University School of Social Work, Universidade Santiago de Compostela, 15704 Santiago de Compostela, Spain; 4 Department of Occupational Therapy, Faculty Padre Ossó, Oviedo University, 33008 Oviedo, Spain; luisjavier@facultadpadreosso.es; 5 Department of Nursing, Physiotherapy and Occupational Therapy, Castilla-La Mancha University, 45600 Toledo, Spain; pedro.moruno@uclm.es

**Keywords:** occupational therapy, community-based participatory research, community service, community health, community development

## Abstract

Background: This review aims to evaluate the level of scientific evidence for the effectiveness of Community Occupational Therapy interventions. Methods: A systematic review was used to analyze and synthesize the studies collected. The databases of Cochrane, OTseeker, OTCATS, Web of Science, Scielo and Scopus were used in order to collect articles published between 2007 and 2020. PRISMA recommendations were followed. Results: A total of 12 articles comprised part of the study (7 randomized controlled studies, 4 systematic reviews and 1 meta-analysis). The main areas of practice were geriatric gerontology (22.1%) and mental health (19.7%), which were statistically significant (χ^2^; *p* < 0.005) compared to the rest. Regarding the studies analyzed, all of them had scores of >7 on the PEDro and AMSTAR scales. Conclusions: Research on Community Occupational Therapy constitutes a consolidated line of research but the objectives and areas of research were limited. Descriptive qualitative methodology predominated and studies on the effectiveness of Community Occupational Therapy interventions showed a medium–low level of evidence.

## 1. Introduction

According to the Ottawa Charter, health is a positive concept that underlines the importance of social and personal resources in order to achieve an adequate state of physical, mental and social well-being. The promotion of health is focused on populations or communities in order to create the necessary conditions for them to improve their health or exercise greater control over it [1,2].

Today, occupational therapists adhere to this perspective, recognizing that health is supported and maintained when individuals are able to engage and participate in occupations and activities at home, school, the workplace and in their community [3]. Occupational therapy actively participates in programs and services to promote the health of communities and populations, developing and implementing occupational-based approaches that pursue the involvement and participation of a population in occupations that promote health in the community [4].

This community perspective of health and its relationship with occupation has given rise to an abundant source of literature in recent years [2,5,6] concerning various theoretical concepts that are proposed as a basis for the practice of Community Occupational Therapy [7,8]. All of these concepts have contributed to the emergence of a new approach to the practice of occupational therapy, emphasizing the promotion of community health as the center of the practice [9]. This approach has been named in various ways: community-based occupational therapy [10], community-centered occupational therapy [9] and Community Occupational Therapy [7,8]. It has been echoed by various models of practice and different institutions [3]. A preliminary review of the scientific literature has allowed us to identify Community Occupational Therapy interventions in different practice settings: primary care [11], geriatrics and gerontology [12], mental health [13], childhood [14] and hospital [15] were among the most relevant.

However, a literature review does not allow us to identify studies that rigorously and clearly describe the definition and characteristics of this type of practice. There are also no systematic reviews of the scientific literature that synthesize the scientific evidence regarding Community Occupational Therapy interventions. Accordingly, we believe that a scoping review is fully justified, since it allows us to delimit and describe an area of evidence regarding Community Occupational Therapy interventions. Only in this way can Community Occupational Therapy represent a solid base for occupational therapist practice, thus moving away from isolated interventions that blur its nature, ignoring the emerging reality that makes it such [16].

Therefore, the research questions guiding this review were: (a) What was the volume, content and characteristics of the research carried out on Community Occupational Therapy? (b) What level of scientific evidence did the analysis of the scientific literature provide on the efficacy of Community Occupational Therapy?

## 2. Materials and Methods

A scoping review method was used to conduct an exploratory mapping of occupational therapy research at the community level. This type of methodology was selected because it allows the examination of a heterogeneous body of knowledge, both in terms of disciplines and research methods [17,18]. In this way, the aim was to identify and synthesize the lines of research explored and identify possible gaps in this research, as well as to elaborate more precise questions in future studies that have the same focus. The review was carried out in five stages [19,20], following the PRISMA-ScR guidelines [17].

First, the research questions were identified based on a preliminary review of the literature. In addition, the MESH terms and keywords of the search were selected in order to identify all available included and/or accessible studies on occupational therapy in the community setting.

Second, a search strategy was carried out using six databases (Cochrane, OTseeker, OTCATS, Web of Science, Scielo and Scopus) with an initial result that located 6453 articles. The MESH terms and keywords used were: “occupational therapy”, “community based”, “community service”, “community-based”, “community health”, “community development”, “community”, and “community-based rehabilitation”. The search was carried out between 1 January 2007 and 1 December 2020 by targeting the MESH terms and keywords in the title, abstract, keywords and main text. The strategy of the search used for Web of Science that was subsequently adjusted to the rest of the databases was: (“Community-Based”) OR (“Community Based”) OR (“Community Service”) OR (“Community Based Rehabilitation”) OR (“Community”) OR (“Community Development”) OR (“Community Health”) AND (“Occupational Therapy”). These were refined by: (Excluding) Types of documents: (Editorial OR Case Report OR Report OR Other OR News OR Retracted Publication OR Abstract OR Letter OR Biography OR Retraction OR Meeting OR Correction OR Reference Material OR Art And Literature OR Unspecified OR Data Paper OR Bibliography Or Book).

Third, the screening process was performed by ordering the references using the Mendeley manager (version 1.5.2), eliminating *n* = 3753 duplicated articles. The following selection criteria were established as inclusion criteria: (a) any study of a quantitative or qualitative nature that looked at occupational therapy in the community setting, (b) all age ranges, (c) studies published in English, Spanish, Portuguese, French and Catalan, (d) any type of population, scope of practice and/or health condition. The exclusion criteria were: those that did not have occupational therapy interventions in the community setting as the main objective of the study. Subsequently, the eligibility process was based on the synthesis of 122 articles.

Fourth, two authors of the study independently performed a complete reading of the 122 selected articles by extracting the data from each article in an Excel table (v.11) prepared by the research team, which recorded the following information from each study: author(s), year of publication, journal, study methodology and design, scope of practice, study objectives or research question, sample, intervention, evaluation tools used in the study, statistical analysis and outcome measurements, limitations and conclusions. Subsequently, a third author independently reviewed the analysis of the extracted data. Any differences in the analysis of the documents between the different authors were resolved by consensus between them. Based on data extracted from the 122 selected articles, the first research question: (a) What was the volume, content and characteristics of the research carried out on Community Occupational Therapy? was answered.

Fifth, the strategy included 40 studies that represented a quantitative synthesis. Furthermore, in order to calculate the level of scientific evidence in the 40 studies, we used the SING scale [21]. With the aim of responding to the second objective of this review—(b) What level of scientific evidence did the analysis of the scientific literature provide on the efficacy of Community Occupational Therapy?—15 studies were selected (randomized clinical trials, systematic reviews, and meta-analysis), due to the fact that these studies were the ones that showed more scientific evidence. Finally, the methodological quality was analyzed in order to determine the extent to which the studies addressed the risk of bias in their designs and analyses. To do this, the PEDro scale [22] was used for the evaluation of randomized clinical trials, and the AMSTAR scale [23] was used for meta-analysis and systematic reviews. Studies that did not exceed a score of at least 50% on the PEDro and AMSTAR scales were discarded. Finally, a total of 12 articles were selected in order to analyze their scientific evidence. Furthermore, with these 40 articles, and to calculate the level of evidence, the SING scale [21] was used.

Finally, a descriptive and inferential statistical analysis of the variables recorded in the study was carried out using IBM SPSS Statistics (version 19) and EPIDAT 3.1. The quantitative variables were expressed by using the mean, frequency and percentage. In the inferential analysis, the Chi-square (χ^2^) test was applied in order to verify the null hypothesis of equality of proportions, using a confidence interval of 95%. This analysis made it possible to identify the volume, content and characteristics of the research and to summarize the existing scientific evidence on occupational therapy interventions in the community setting [19].

## 3. Results

**Figure 1:**
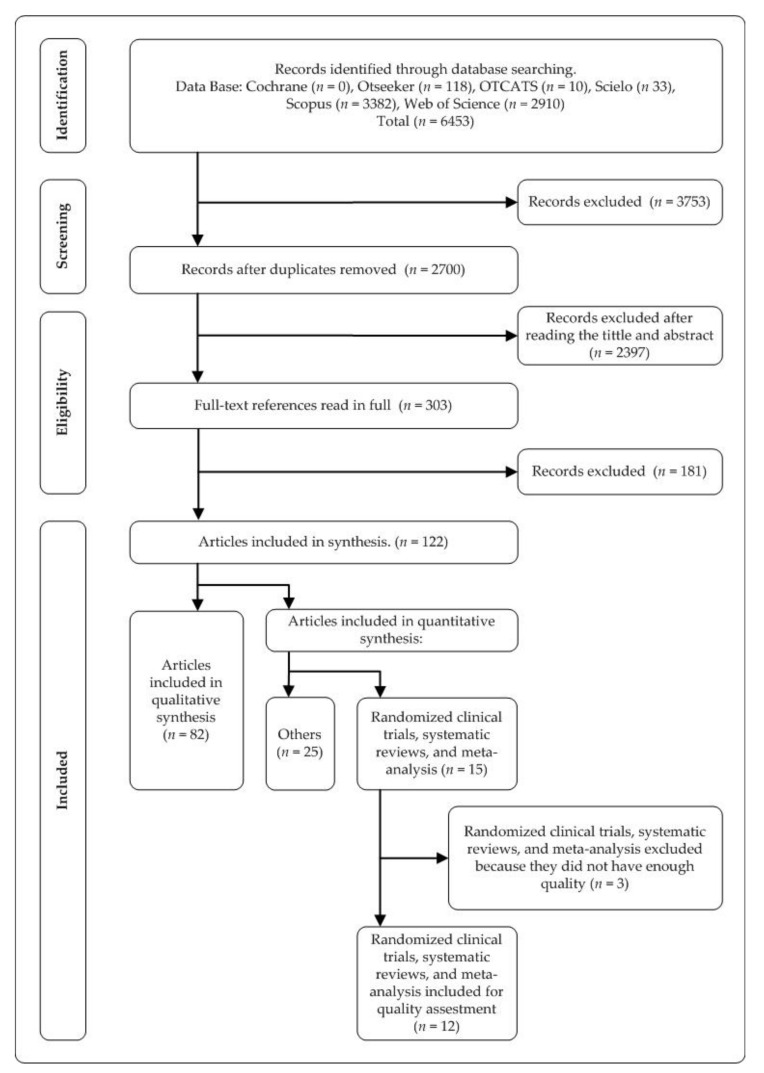
Flow chart diagram.

The search identified 122 relevant documents after a full-text review (Figure 1).

### 3.1. Research synthesis

#### 3.1.1. Research volume

**Figure 2:**
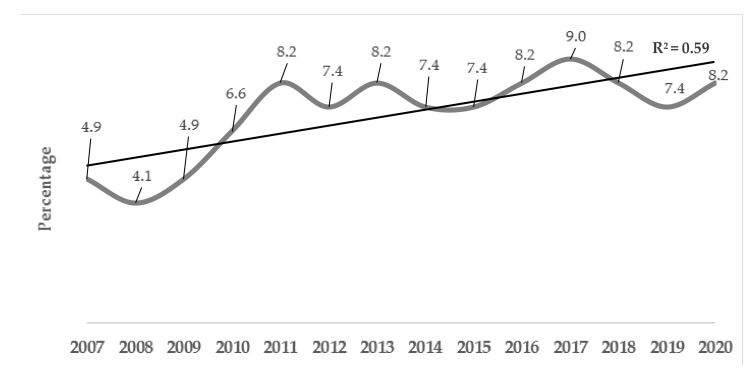
Temporal evolution of articles published between 1 January 2007 and 1 December 2020.

The 122 articles included in the synthesis were published in 49 indexed journals, primarily in English. They were predominantly publications in occupational therapy journals: Australian Occupational Therapy Journal *n* = 15 (12.3%); American Journal of Occupational Therapy *n* = 13 (10.7%) and the Scandinavian Journal of Occupational Therapy *n* = 10 (8.2%). The rest of the journals published less than ten articles that were relevant to the research questions between 2007 and 2020. As shown in Figure 2, the number of publications fluctuated, although a certain regularity was maintained, with an average of 8.3 annual publications. Furthermore, a gradual and stable increase in published studies was observed in the last ten years (Figure 2).

The countries that produced the most literature on the objectives of the study were: USA *n* = 40 (32.8%), Australia *n* = 25 (20.5%), Canada *n* = 24 (19.7%) and England *n* = 10 (8.2%). The rest of the countries had less than ten published articles. Only one study was found in Spanish Puerto Rico *n* = 1 (0.8%) and one in Portuguese Brazil *n* = 1 (0.8%).

#### 3.1.2. Content of the Research

The practice areas with the greatest number of investigations were: geriatrics and gerontology *n* = 27 (22.1%), mental health *n* = 24 (19.7%), and physical dysfunction (*n* = 8; 6.6%). In the rest, the percentage number of studies was less than 6%. In the areas of geriatrics and gerontology, and mental health, a statistically significant difference was identified (χ^2^; *p* < 0.005) when compared to the rest of the practice areas. Regarding the research objectives, those related to the assessment of evaluation tools and the application of practice models *n* = 18 (14.8%), the evaluation of intervention programs to improve health *n* = 12 (9.8%), and the evaluation of leisure interventions and/or social participation in the community *n* = 12 (9.8%), were the most frequent. The average duration of the intervention programs evaluated was 2.5 months, and the rest of the research objectives were less than 9.8%.

#### 3.1.3. Characteristics of the Research

Qualitative research predominated in the study. When compared with the rest of the methodological strategies, a statistically significant difference (χ^2^; *p* < 0.005) was identified. A total of 58.2% of the studies used a qualitative design: 20(16.4%) phenomenological, 18 (14.8%) participatory action research (PAR), 15 (12.3%) ethnographic, 12 (9.8%) narrative, and 6 (4.9%) meta-ethnographies. A total of 32.7% of the studies used a quantitative design: 7 (5.7%) RCT, 7 (5.7%) systematic reviews, 1 (0.8%) meta-analysis, 3 (2.5%) pilot studies, 7 (5.7%) case–control studies and 15 (12.3%) cohort studies.

#### 3.1.4. Quality of the Evidence

Quality analysis of the randomized controlled studies, systematic reviews and meta-analyses revealed a mean and mode score of 6, median of 6.9 and a range of 5.9–8.

Of the 15 studies initially identified, only three [24,25,26] did not exceed 50% of the scores on the PEDro or AMSTAR scales (Table 1). According to the items of the different checklists used, the following were assessed: the source of the evidence and its characteristics, the nature of the findings reported, statistical analysis, the internal and external validity of the designs, the strength of association between the variables, the risk of bias across the studies, the measurement tools across the studies, and the checklist results.

Concerning the quantitative aspects of the scientific evidence, the studies scored with 1- and 2- predominated, compared to 1++ and 1+ (Table 2).

It should be noted that due to the heterogeneity and methodological limitations of the studies, it was not possible to perform a meta-analysis with the research results, nor perform detailed comparisons between studies. Table 3 provides a summary of the studies included. In the presentation of this summary, the following have been detailed: thematic areas, objectives, characteristics (duration of the study, population profile, number of participants or number of articles included in the reviews), results, and conclusions of the investigations carried out on the efficacy of Community Occupational Therapy interventions that exceeded the minimum criteria of rigor and scientific quality (scores ≥7 on the PEDro and AMSTAR scales).

## 4. Discussion

Regarding the first question of this study, from 2007 to the present, the number of studies on Community Occupational Therapy experienced a gradual increase, which may indicate growing interest in this area of research. Principally, this fact may be related to an aging population, and that there needs to be more outcome studies in order to evaluate the effectiveness of such an intervention [37,38,39]. In relation to this fact, we could confirm that research on Community Occupational Therapy currently constituted a consolidated line of research during the period studied.

According to the data obtained, it appeared that research in areas of geriatrics and mental health concentrated most of the research (exceeding 50% of the articles selected). In addition, the main objective of a quarter of the research in these areas focused on the evaluation of the effectiveness of different intervention programs. In geriatrics, such programs had the main objectives of improving the functionality and quality of life, reducing the risk of falls and overloading caregivers, increasing autonomy in the performance of activities of daily living and independence in the home, and promoting the health and well-being of healthy older people residing in the community.

In the case of mental health, the main objective of intervention programs is to improve the performance of basic activities of daily life, provide independence in the community, social participation, quality of life, mood and general health, as well as reducing addiction relapse and caregiver burden. However, the average duration of such programs was short, with an average of 2.5 months, which considering the objectives, is usually achievable in the medium- or long-term once such programs are established [40]. In addition, the focus is on individual interventions within the community, to the detriment of actions aimed at promoting the health of communities and populations, distancing itself from the guidelines that direct the practice of occupational therapy in the community [3]. Surprisingly, the little research undertaken on interventions for health promotion and disability prevention has traditionally been linked to community health.

Therefore, in light of the results of this study, we advocate increased research on health promotion and prevention of disability in the community, with the aim of expanding the scientific evidence on the efficacy of Community Occupational Therapy related to these spheres.

Regarding the methodological characteristics of the research, on the one hand, we considered that the range of research objectives was limited. This circumstance could be related to the meagre experience and poor tradition of the practice of Community Occupational Therapy, which implies a significant lack of tools and intervention strategies, as well as the necessary skills for the implementation of distinctive actions and proven effectiveness [4,41]. In this regard, we fully agree with the numerous authors [41,42,43] who have advocated the diversification of study objectives and the development of lines of research that make it possible to gather scientific evidence on the efficacy of the practice of Community Occupational Therapy.

On the other hand, it should be noted that the qualitative methodology of a descriptive nature predominated, since the percentage of quantitative studies (32.79%) was significantly lower than the percentage of qualitative studies (58.20%). This fact could be related to the suitability of this methodology, in relation to the objectives usually proposed in Community Occupational Therapy studies, which seek to apprehend the subjective experience of the health of members of the community. Considering the data obtained in this review, it appeared that research on Community Occupational Therapy has reached a period of consolidation, adopting a variety of both qualitative and quantitative approaches, although qualitative studies still predominated. However, we believe that it would be advisable to increase quantitative research in order to provide scientific evidence [44,45,46].

Nevertheless, it should also be noted that, according to the data analyzed in this scoping review, some of the studies identified had low methodological quality. Therefore, we consider it necessary to improve such quality. These findings seemed to indicate the need to improve the quality of evidence from the effects of Community Occupational Therapy programs in specific areas, in order to reduce the variability of the practice and improve its efficacy [47,48]. It should also be noted that research on Community Occupational Therapy has been carried out mainly in Anglo-Saxon countries. This circumstance could generate a possible bias in research on the selection procedure of the study population [49,50,51,52]. Therefore, we advocate increasing the number of countries in which this study objective is investigated, in order to collect information on the social and cultural particularities of the practice of Community Occupational Therapy.

Regarding the second question of this review, despite the fact that in recent years there has been a significant increase in evidence-based research as a fundamental basis for the best choice of occupational therapy practice in the community, the quality of evidence of efficacy for this practice is inconclusive or sparse. A high percentage of studies based on the efficacy of Community Occupational Therapy interventions showed a medium–low level of evidence. Therefore, not all the scientific studies analyzed had the same value with regard to decision making in choosing the best available practice. In fact, studies classified as 1- and 2- should not be used in the recommendation-making process due to their high potential for bias. However, it should be considered that the studies included were too small to detect this effect. It is possible that methodological limitations and the heterogeneity of the studies included meant that the effect was not detected.

In this same sense, it should be noted that, in the field of mental health, despite the high percentage of studies identified in the review, no randomized controlled studies, meta-analyses or systematic reviews have been carried out. Therefore, the scientific evidence gathered regarding the efficacy of occupational therapy interventions in this setting is sparse.

Along the same lines, due to the analysis carried out in this scoping review, we should consider the apparent contradiction between the characteristics of interventions considered as Community Occupational Therapy and the definition of this area in the specialized literature on occupational therapy.

In recent years, occupational therapy has suggested a profound transformation of the perspective toward the concept of health that changes from the individual to the community [53,54,55], which has been echoed by various models of practice [56] and diverse institutions [3]. From this new perspective, according to Wilcock and Townsend [42]: “[…] it is not only about reducing illness and disability in individuals […] but about promoting a broad notion of health, understood as the ability and opportunity to live, work and play in safe communities that provide support”. In accordance with these guiding principles, Community Occupational Therapy stands as a paradigm of this change in a health perspective [7,57,58].

However, the results of this review show that scientific research on Community Occupational Therapy focuses on specific groups (mainly geriatrics and mental health), with time-limited interventions, which are fundamentally based on an individual concept of health. This circumstance could be related to the absence of a clear and precise definition of the notion and scope of Community Occupational Therapy [41].

From our point of view, the absence of this precise definition, as well as the health exegesis that accompanies it, can lead to the practice of Community Occupational Therapy based on short-term interventions, centered on individuals residing “within” the community, as the results of this scoping review seemed to show.

Therefore, we advocate for the practice of Community Occupational Therapy that implies a profound change in the intervention perspective, based on occupational justice and empowerment, which requires medium- and long-term interventions “in, with and from” the community. In other words, Community Occupational Therapy should understand the community as a unit of analysis and independent intervention [58,59]. Only in this way will we be able to modify the conditions that allow the community to carry out and engage in occupations that ultimately promote the health and well-being of its members.

In short, we defend a greater precision and clarity in the definition of the notion of Community Occupational Therapy, the ultimate support for a real change in the practice of our profession in this area.

### 4.1. Limitations of the Research

A detailed analysis of the methods used to assess the risk of bias in the studies included in this review was beyond the scope of this study. Therefore, the absence of this information can be considered as a limitation of the study.

### 4.2. Recommendations for Future Research

Improving the methodological quality of research in this area is a basic recommendation for increasing the scientific evidence on the efficacy of Community Occupational Therapy interventions [50,51].

It is advisable to develop research projects that allow scientific evidence to be gathered on the efficacy of Community Occupational Therapy interventions in the field of mental health.

## 5. Conclusions

The efficacy of occupational therapy practice in the community is not entirely clear, but these interventions appear promising and deserve further investigation. The quality of evidence on the effects of Community Occupational Therapy programs is inconclusive or sparse. The Community Occupational Therapy interventions to reduce the risk of falls and enhance the performance of activities of daily life in older people seem to be the most effective strategies. Research on Community Occupational Therapy must heighten the methodological quality of research in order to reduce the variability of the practice and improve its efficacy.

## Figures and Tables

**Table 1:** Methodological quality.

Reference	Journal and Country	Scale									
		**1**	**2**	**3**	**4**	**5**	**6**	**7**	**8**	**9**	**10**
Randomized Controlled Studies		PEDro									
Garvey et al. [11]	BMC Fam Pract (UK)	✔	✔	✔	**✕**	**✕**	**✕**	**✕**	✔	✔	✔
Clark et al. [12]	J Epidemiol Community Health (UK)	✔	**✕**	✔	**✕**	**✕**	✔	**✕**	✔	✔	✔
Graff et al. [27]	BMJ (UK)	✔	✔	✔	**✕**	**✕**	✔	✔	✔	✔	✔
Lam et al. [28]	Int J Geriatr Psychiatry (UK)	✔	✔	✔	**✕**	**✕**	✔	✔	✔	✔	✔
Graff et al. [29]	J Gerontol A Biol Sci Med Sci (USA)	✔	✔	✔	**✕**	**✕**	✔	**✕**	**✕**	✔	✔
Hirsch [30]	BMJ Evid Based Med (UK)	✔	**✕**	**✕**	**✕**	**✕**	✔	**✕**	✔	✔	✔
Ciaschini et al. [31]	Age Ageing (UK)	✔	**✕**	✔	**✕**	**✕**	**✕**	✔	✔	✔	✔
Systematic review		AMSTAR									
De Coninck et al. [32]	J Am Geriatr Soc (USA)	✔	✔	✔	✔	**✕**	✔	✔	✔	✔	✔
Hall and Skelton [33]	Br J Occup Ther (UK)	**✕**	✔	✔	✔	✔	✔	✔	**✕**	**✕**	**✕**
Tate et al. [34]	Brain Impair (Australia)	**✕**	✔	✔	✔	✔	✔	✔	**✕**	**✕**	✔
Parente et al. [35]	Occup Ther Int (UK)	✔	**✕**	✔	✔	✔	✔	**✕**	**✕**	**✕**	**✕**
Meta-analysis		AMSTAR									
Clemson et al. [36]	J Aging Health (USA)	✔	✔	✔	✔	✔	✔	✔	✔	✔	✔

✔: Meets the criteria; **✕**: Does not meet the criteria. PEDro—1: The selection criteria were specified; 2: subjects were randomized in groups (in a crossover study, subjects were randomized as they received treatments); 3: allocation was hidden; 4: the groups were similar at the beginning in relation to the most important prognostic indicators; 5: all subjects were blinded; 6. all therapists who administered the therapy were blinded; 7: all raters who measured at least one key outcome were blinded; 8: measurements for at least one of the key outcomes were obtained from more than 85% of the subjects initially assigned to the groups; 9: results were presented for all subjects who received treatment or were assigned to the control group, or where this could not be, data for at least one key outcome were analyzed by “intention to treat”; 10. results from statistical comparisons between groups were presented for at least one key outcome. AMSTAR (Assessing the Methodological Quality of Systematic Reviews)—1: Was the design provided a priori?; 2: was there a selection of duplicate studies and data extraction?; 3: was an exhaustive search of the literature carried out?; 4: was publication status (i.e., gray literature) used as an inclusion criterion?; 5: was a list of studies (included and excluded) provided?; 6: were the characteristics of the included studies provided?; 7: was the scientific quality of the included studies assessed and documented?; 8: was the scientific quality of the included studies used appropriately to formulate conclusions?; 9: were the methods used to combine the results of the studies appropriate?; 10: was the probability of publication bias assessed?

**Table 2:** Levels of evidence and grades of recommendation according to SIGN.

	1++	1+	1-	2++	2+	2-	3	4
Cohort studies					8	7		
Case–control studies					2	5		
Pilot studies		1	2					
Meta-analysis	1							
Systematic review	1	2	4					
RCT		2	5					
Total	2	5	11	0	10	12	0	0

RCT: randomized controlled studies; 1++: meta-analysis, systematic reviews of clinical trials or high-quality clinical trials with very little risk of bias; 1+: meta-analysis, systematic reviews of clinical trials or well conducted clinical trials with little risk of bias. 1-: meta-analysis, systematic reviews of clinical trials or clinical trials with high risk of bias; 2++: systematic reviews of cohort or case–control studies or high-quality diagnostic test studies, cohort or case–control studies of high-quality diagnostic tests with very low risk of bias and with a high probability of establishing a causal relationship; 2+: cohort or case–control studies or well-conducted diagnostic test studies with low risk of bias and with a moderate probability of establishing a causal relationship; 2-: cohort or case–control studies with; 3: Non-analytic studies, e.g. case reports, case series; 4: expert opinion.

**Table 3:** Synopsis of findings for randomized controlled studies, systematic reviews and meta-analyses.

Population and Sample	D *		Objective		Intervention Type	Assessment Tools and Results	MQ **
Geriatrics and Gerontology							
Randomized Controlled Studies							
Graff et al. (2006) [27]. Netherlands							8/10
135 people ≥ 65 years old with mild-moderate dementia.	12		To evaluate the efficacy of community-based occupational therapy intervention in improving the daily functioning of patients.		Ten occupational therapy sessions (cognitive and behavioral) to train patients (use of aids, compensate for cognitive impairment) and caregivers (coping and supervisory behaviors).	Assessment of Motor and Process Skills (AMPS), and Daily Activities in Dementia (IDDD). Sense of Competence Questionnaire (SCQ). There was a significant pre- and post-improvement in patients and caregivers in the intervention group compared to the control group (the differences were 1.5 (95% confidence interval 1.3 to 1.7) for the AMPS; −11.7 (−13.6 to −9.7) for the IDDD and (11.0; 9.2 to 12.8) for SCQ. The number of patients needed to treat in order to achieve a clinically relevant improvement in motor skills score was 1.3 (1.2 to 1.4) at six weeks, whereas those who received occupational therapy performed significantly better. In ADL those compared to those who did not at 12 weeks showed this improvement was still significant (effect sizes 2.7, 2.4 and 0.8).	
Lam et al. (2010) [28]. Hong Kong							8/10
102 people ≥ 65 years old with mild dementia, residents in the community.	16		To evaluate whether occupational therapy interventions focused on case management alleviated the burden on the caregiver and improved the quality of life for people with dementia.		Case Management.	Primary outcome: Zarit Burden Scale (ZBI). General Health Questionnaire (GHQ). Personal Well-Being Index for Adults (PWI-A). Secondary outcome: Mini Mental State Examination (MMSE). Neuropsychiatric Inventory (NPI). Cornell Scale for Depression in Dementia (CSDD). Personal Well-Being Index for the Intellectually Disabled (PWI-ID). The use of day centers and home assistants was higher in the case management group, both in the fourth and twelfth month of follow-up (*p* < 0.005). The study showed significant effects in reducing the burden on the caregiver.	
Graff et al. (2007) [29]. Netherlands							6/10
135 couples of patients older than 65 years with mild or moderate dementia and their caregivers.	5		To evaluate the effectiveness of a multidisciplinary community program aimed at optimizing the management of cases with risk of fractures related to falls.		Ten sessions of cognitive and behavioral occupational therapy.	Diabetes Quality of Life (DQOL). Cornell Scale for Depression (CSD). Center for Epidemiologic Studies Depression Scale (CES-D). General Health Questionnaire (GHQ-12). This study was a replica of the study by Graff et al. [26], which corroborated the results of the previous study. The improvement in the COD of patients in general (0.8, 95% confidence interval (CI) 0.6–0.1, effect size 1.3) and the COD of the caregivers in general (0.7, 95% CI 0.5–0.9, effect size 1.2) was significantly better in the intervention group compared to controls. Scores on other assessment instrument measurements and their outcome also improved (*p* < 0.007 with Bonferroni correction). Improvement also obtained at 12 weeks. Community occupational therapy improved mood, quality of life, health status and caregivers’ sense of control, and was recommended for patients with dementia and caregivers.	
Hirsch (2007) [30]. Netherlands							6/10
135 people ≥ 65 years old (56% women) with mild-moderate dementia and residents of the community.	5		To evaluate the efficacy of community-based occupational therapy interventions in the daily functioning of older patients with dementia and in the competence of caregivers.		Ten occupational therapy sessions using client-centered guidance to modify the patient environment, ADL performance, and training of caregivers in maintaining patient autonomy and their own social participation.	Ten one-hour sessions of occupational therapy were conducted in homes (*n* = 68) together with the same number of sessions without occupational therapy intervention (*n* = 67). The study showed a statistically significant improvement (*p* = 0.005) in daily functioning in patients and in the competence of their caregivers in the group that received the occupational therapy intervention.	
Ciaschini et al. (2009) [31]. USA							6/10
201 people ≥ 55 years old at risk of hip fracture due to falls.	48		To evaluate the effectiveness of a multidisciplinary community program to optimize the management of cases with risk of fractures.		Components of the intervention included assessment of risk of falls, functional status and family environment, and patient education.	Compared with usual care, the intervention increased the number of referrals to physical therapy (21% (21/101) vs. 6.0% (6/100); relative risk (RR) 3.47, confidence interval (CI) 95% 1.46–8.22) and occupational therapy (15% (15/101) vs. 0%; RR 30.7, 95% CI 1.86 to >500), but it did not reduce the risk of falls since at 12 months, those in the intervention group were higher than in the usual care group (23% (23/101) vs. 11% (11/100); RR 2.07, 95% CI 1.07–4.02).	
Clark et al. (2011) [12]. USA							6/10
460 people (60-95 years old) in the Los Angeles metropolitan area (USA).	24		To determine the efficacy and economic profitability of occupational therapy and health promotion intervention in community-residing elderly people.		Monthly outings to the community were programmed to facilitate direct experience with the content of the intervention, such as the use of public transport.	The participants of the intervention, in relation to the control group, showed improvement in scores of vitality indices, social functioning, mental health, compound mental functioning, and satisfaction with life, as well as a decrease in depressive symptoms and body pain (*p* < 0.005). Furthermore, it was economically profitable when comparing occupational therapy intervention costs with other alternative interventions.	
Systematic review							
De Coninck et al. (2017) [32]. Netherlands							9/10
Nine studies up to 2015 with a population of 3163 people ≥ 60 years of age with chronic disabilities residing in the community.	-		To evaluate the efficacy of Community Occupational Therapy interventions in improving performance of activities of daily living.		-	A significant increase in performance improvement was identified, with a standardized mean difference of 0.30 in the case of activities of daily living (95% CI 0.50 to 0.11; *p* = 0.002); 0.44 in the case of social participation activities (95% CI 0.69 to 0.19; *p* = 0.007) and 0.45 in the case of mobility in the community (95% CI 0.78 to 0.12; *p* = 0.007).	
Hall and Skelton (2012) [33]. United Kingdom							6/10
17 studies published between 1999 and 2010 with 586 people with dementia and their caregivers.	-		To identify the efficacy of occupational therapist interventions to increase support for caregivers of people with dementia.		-	There was an increase in all variables related to the support perceived by the caregiver, except for one related to knowledge of the disease.	
Meta-analysis							
Clemson et al. (2008) [36]. Australia							10/10
3298 people ≥ 65 years old who resided in the community.	-		To determine the efficacy of occupational therapy interventions in local community services for reducing the risk of falls in older people.		-	The collected analysis of the six clinical trials (*n* = 3298) showed a total reduction of 21% in the risk of falls (RR = 0.79, 95% CI = 0.65 to 0.97). Pooled analysis of four clinical trials with participants having a high risk of falls (*n* = 570) showed an absolute risk difference of falling of 26% and a clinically significant reduction of 39% in falls (RR = 0.61, 95% CI 0. 47 to 0.79).	
Physical dysfunction							
Systematic review							
Tate et al. (2014) [34]. Australia							7/10
Articles: Medline (since 1946), PsycINFO (since 1806), and PsycBITE (since 1806), to 2014. Nine studies and a population of 132 adults with traumatic brain injury, residents in the community.		-		To identify and evaluate the efficacy of community-based occupational therapy interventions for the improvement of leisure/social activity after suffering a head injury.	-	A total of 58 statistical comparisons were made, but only 25 (43%) were significant. The effect size for improvement in the experimental group was small.	
Primary care							
Randomized controlled studies							
Garvey et al. (2015) [11]. Ireland							6/10
50 people with problems associated with the management of multimorbidity and chronic conditions.	-		To evaluate efficacy, increased frequency of participation in community activities, improvement of quality of life and independence of ADL.		OPTIMAL. Occupational Therapy Led Self-Management Support Programme (six weeks).	There was an increase in the frequency of participation in activities within the community (*p* < 0.001), in the subjective perception of performance and personal satisfaction.	
Natural disasters							
Systematic review							
Parente et al. (2017) [35]. Italy.							5/10
Ten studies published between 2005 and 2015	-		To evaluate the available evidence on the efficacy of occupational therapist interventions in disaster situations.		Articles on rehabilitation and occupational therapy interventions in disaster management (after earthquakes) were included.	Insufficient scientific evidence and scarcity of studies in the literature. The importance of access to rehabilitation interventions, including a rehabilitation team and providing methods to address difficult evacuations.	

D *: Duration in weeks; MQ: Methodological quality. ** The systematic review and meta-analysis were evaluated with AMSTAR. The randomized controlled studies were evaluated with PEDro.

## Data Availability

No data availability statement.
